# An Accurate, Flexible and Small Optical Fiber Sensor: A Novel Technological Breakthrough for Real-Time Analysis of Dynamic Blood Flow Data *In Vivo*

**DOI:** 10.1371/journal.pone.0114794

**Published:** 2014-12-31

**Authors:** Qiao-ying Yuan, Ling Zhang, Dan Xiao, Kun Zhao, Chun Lin, Liang-yi Si

**Affiliations:** 1 Department of Geriatrics, Southwest Hospital, the Third Military Medical University, Chongqing, PR China; 2 Department of Out-patient clinic (OPD), Southwest hospital, the Third Military Medical University, Chongqing, PR China; 3 School of Physics and Mechanical & Electrical Engineering, Xiamen University, Xiamen, PR China; Washington State University, United States of America

## Abstract

Because of the limitations of existing methods and techniques for directly obtaining real-time blood data, no accurate microflow *in vivo* real-time analysis method exists. To establish a novel technical platform for real-time *in vivo* detection and to analyze average blood pressure and other blood flow parameters, a small, accurate, flexible, and nontoxic Fabry-Perot fiber sensor was designed. The carotid sheath was implanted through intubation of the rabbit carotid artery (n = 8), and the blood pressure and other detection data were determined directly through the veins. The fiber detection results were compared with test results obtained using color Doppler ultrasound and a physiological pressure sensor recorder. Pairwise comparisons among the blood pressure results obtained using the three methods indicated that real-time blood pressure information obtained through the fiber sensor technique exhibited better correlation than the data obtained with the other techniques. The highest correlation (correlation coefficient of 0.86) was obtained between the fiber sensor and pressure sensor. The blood pressure values were positively related to the total cholesterol level, low-density lipoprotein level, number of red blood cells, and hemoglobin level, with correlation coefficients of 0.033, 0.129, 0.358, and 0.373, respectively. The blood pressure values had no obvious relationship with the number of white blood cells and high-density lipoprotein and had a negative relationship with triglyceride levels, with a correlation coefficient of –0.031. The average ambulatory blood pressure measured by the fiber sensor exhibited a negative correlation with the quantity of blood platelets (correlation coefficient of −0.839, P<0.05). The novel fiber sensor can thus obtain *in vivo* blood pressure data accurately, stably, and in real time; the sensor can also determine the content and status of the blood flow to some extent. Therefore, the fiber sensor can obtain partially real-time vascular rheology information and may thus enable the early diagnosis of blood rheology disorders and diseases.

## Introduction

Many diseases are associated with abnormal blood flow, including atherosclerosis, diabetes, hyperlipidemia, coronary heart disease, and peripheral vascular disease. However, early detection of micro flow lesions is difficult because blood flow is constantly changing. Testing of blood samples directly through vein hemospasia and indirectly through in vitro methods can only reflect data at one specific time, which is inaccurate. The vasculopathy of microvessels is latent and difficult to determine through normal clinical examination [1], [2]. The existing Doppler method is limited to the testing of large- and medium-sized vessels and is affected by blood flow angle and depth, leading to inaccuracy in single blood drawings and in vitro Doppler detection. Because of the limitations of the existing methods and techniques for obtaining real-time blood data directly, no accurate microflow in vivo real-time analysis method exists. Therefore, overall real-time microvessel rheology data cannot be obtained and pathophysiological conditions cannot be understood. By utilizing interdisciplinary advantage, we break limitations in current research methods to realize the acquisition and analysis of in vivo dynamic blood information based on a new optical fiber sensor system. We also realize the basic principle of blood flow from the whole biology level, provide a basis for researching various early-phase microangiopathies and exploring new therapeutic targets from a new perspective, and utilize new methods. Thus, in the present study, an optical fiber sensor is designed for using as a flat and round membrane with a hard center structure that is very easy to operate, is reliable, can be processed and controlled, possesses a diameter of 1.4 mm, and can enter small vessels. The fiber sensor has the advantages of small size, accuracy of measurement, flexibility, and nontoxicity [3]–[5].

## Methods

### 1 Development of fiber sensor

A small-range pressure sensor was constructed to obtain the blood pressure parameters of animals. The pressure sensor was used to design a diaphragm structure with a target range from −40 kPa to 80 kPa.

### 2 Production design of the cavity

**Figure 1:**
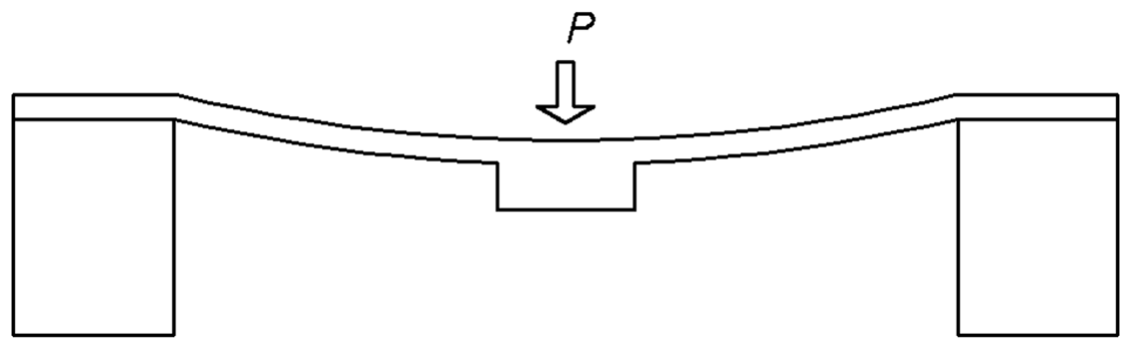
Flat diaphragm structure with a hard center.

The diaphragm structure of the pressure sensor was generally designed to be a flat diaphragm structure with a hard center, as illustrated in Fig. 1.

The characteristic equation for the structure is



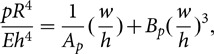
(1–1)where
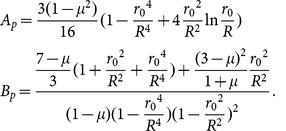
(1–2)


The maximum bending stress is concentrated on the edges of the diaphragm and on the edges of the hard center, the value of which is 



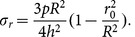
(1–3)


The pressure-deflection curve can be obtained through numerical simulation. The curve exhibits great nonlinearity, and the maximum deflection at the center is 



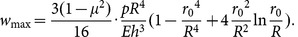
(1–4)


**Figure 2:**
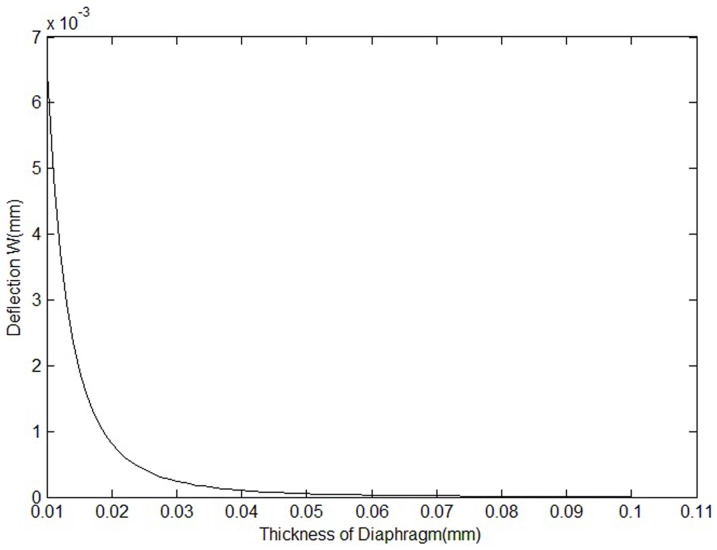
Correlation of maximum deflection and thickness of the diaphragm.

Fig. 2 shows the correlation of the maximum deflection and the thickness of the diaphragm.

Although the deflection of the diaphragm is much smaller than the thickness of the diaphragm, the nonlinearity of the diaphragm deformation will be under quantitative calculation when the sensor is designed. The thickness of the diaphragm is assumed to be 20 µm. Its characteristic equation obtained using the large defection theory is 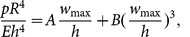
(1–5)


**Figure 3:**
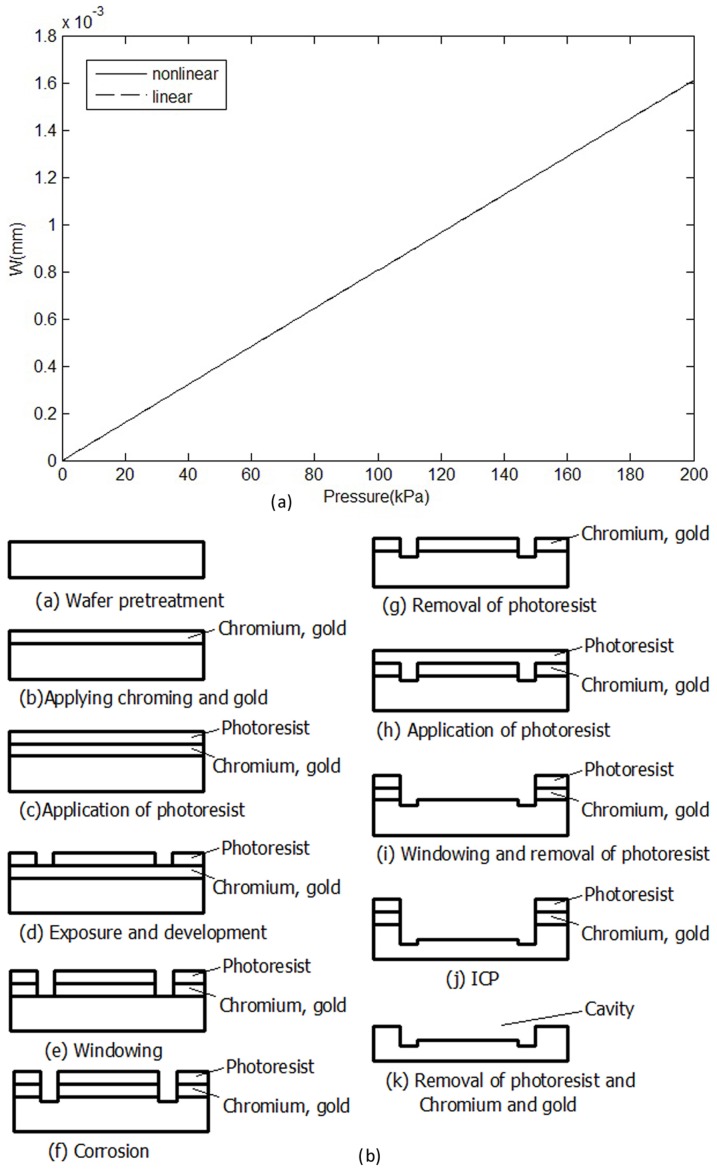
Production design of the cavity. (a) Correlation of deflection and pressure. (b)Production process of the diaphragm.

According to the characteristic equation, the deflection-pressure curve is drawn, as shown in Fig. 3-a.

Based on Fig. 3-a, the nonlinearity of the diaphragm deformation is very small. The nonlinearity is less than 0.01%F.S. according to numerical simulation.

According to the frequency response formula, the lowest resonant frequency of the diaphragm is 



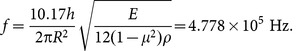
(1–6)


Pretreatment mainly included washing, drying, and annealing. For the metal evaporation mask, gold was used because it is relatively chemically stable and thus an ideal material for glass etching masks. To improve the adhesion of the gold mask layer and prevent the glass from falling off, a Cr layer was added between the glass and gold layers. For the spin coating of the photoresist, exposure and development, windowing, corrosion, photoresist removal, chromium and gold removal, et al, the steps indicated in Fig. 3-b were used.

### 3 Determiningthe real-time blood flow velocity of living animals

The experimental protocols were approved by the Animal Experimentation Ethic Committee (the Third Military Medical University (TMMU), Chongqing, China). All experimental procedures conformed to the Guide for the Care and Use of Laboratory Animals published by the US National Institutes of Health (NIH publication 85-23, revised 1996). The animals were used to establish methods and explore their feasibility. The common carotid arteries of eight rabbits were separated under anesthetic conditions. Then, the artery-sheathing canal was inserted after heparinization and connected with the optical fiber sensor and optical fiber interrogator to obtain blood flow information.

### 4 Color Doppler ultrasound test method

The color Doppler ultrasound diagnostic apparatus used was a Siemens Suqoia512, with a transducer frequency of7.5 MHz to 10 MHz. The rabbits' neck fur was removed and applied using ultrasound glue to identify the bilateral carotid arteries. Longitudinal scanning was conducted from the beginning of the bilateral carotid artery. The common carotid artery was displayed to measure its inner diameter and to observe whether plaque formed on the blood vessel wall and cavity. During plaque testing, the plaque size was measured. The blood vessel cavity was also examined to determine if it was too narrow. Finally, the Doppler method was used to determine the fullness of color, blood flow, and pattern of the blood flow frequency spectrum.

### 5 Determining changes in blood pressure using a multi-channel physiological recorder

The sheathing canal was implanted in the rabbits' carotid arteries after heparinization and connected with the pressure sensor of the multi-channel physiological recorder, thus allowing blood pressure measurement.

### 6 Checking of blood biochemistry and blood viscosity

After disinfection, fresh blood from the rabbits' ears was collected to test for changes in blood viscosity. After the experiment, 4 ml of the rabbits' blood was extracted for centrifugation and blood fat examination.

### 7 Statistical analysis

The data are expressed as the means ± standard deviation. Analysis of variance (SPSS 14.0 statistical software, SPSS, Inc., Chicago, IL, USA) was used to assess the data. P<0.05 was considered statistically significant. Viability was compared across multiple experimental groups using a one-way analysis of variance (ANOVA) followed by post-hoc Tukey analysis to determine significant differences (P<0.05) between the different groups. Relationships between continuous variables were examined using simple linear regression analyses. Non-normally distributed data were log-transformed before analysis.

## Results

### 1 Cavity design

The residual in the crystal glass was eliminated by annealing the glass. After annealing in 380°C for two hours, aliasing had obviously been eliminated. The diameter of the optical fiber sensor was 1.4 mm.

### 2 Anodic bonding process

**Figure 4:**
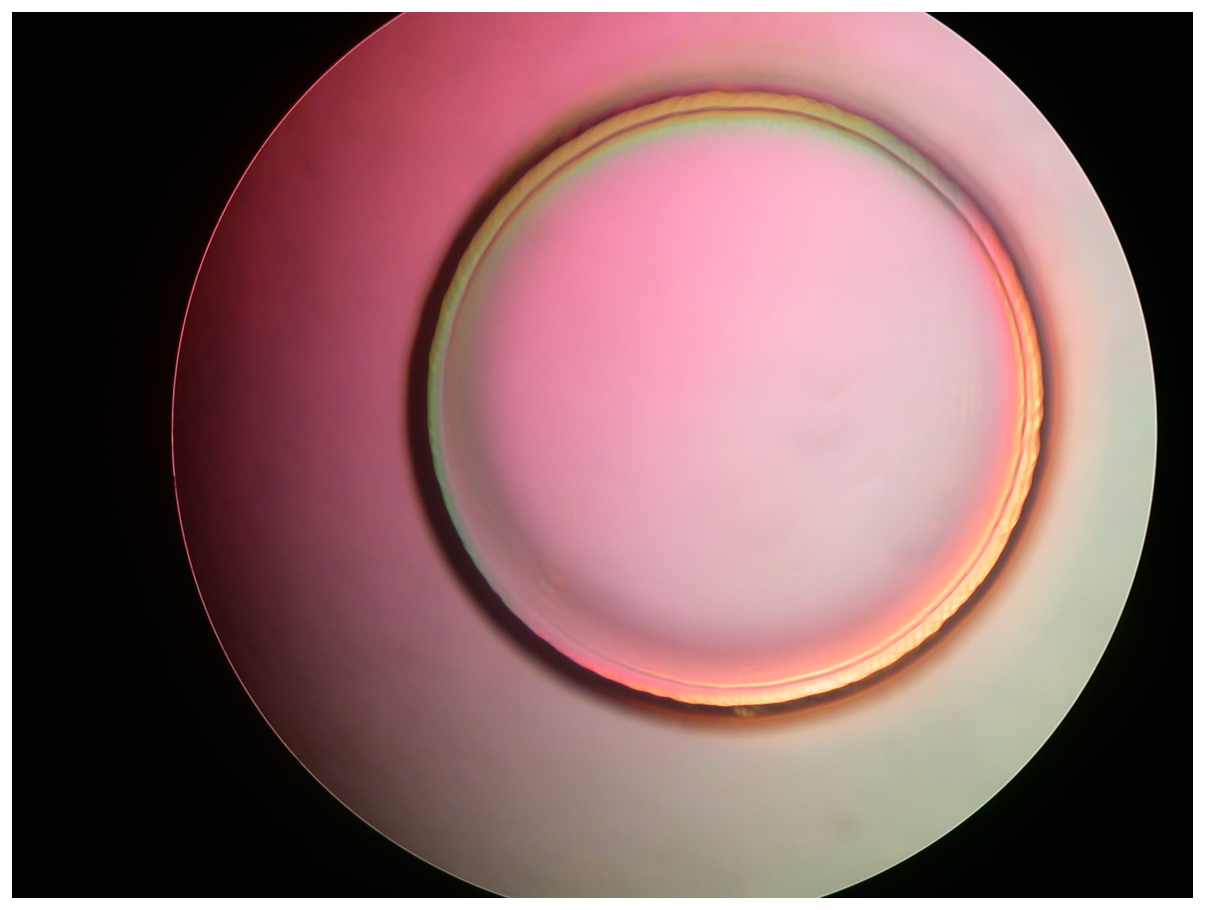
Bonding design sketch.

A vacuum-bonding machine was utilized to input appropriate voltage and pressure parameters under bonding conditions in vacuum. The bonding design without hard center for testing is shown in Fig. 4, in which the gray white area in the outer ring is the bonding area. A tensile test indicated that the bond strength was approximately 12 MPa.

### 3 General condition of animals

The anesthetic effect of sodium amytal in the animals was good. Their vital signs were stable during the entire experiment. Some of the animals required additional anesthetic.

### 4 Experiment on living animals

**Figure 5:**
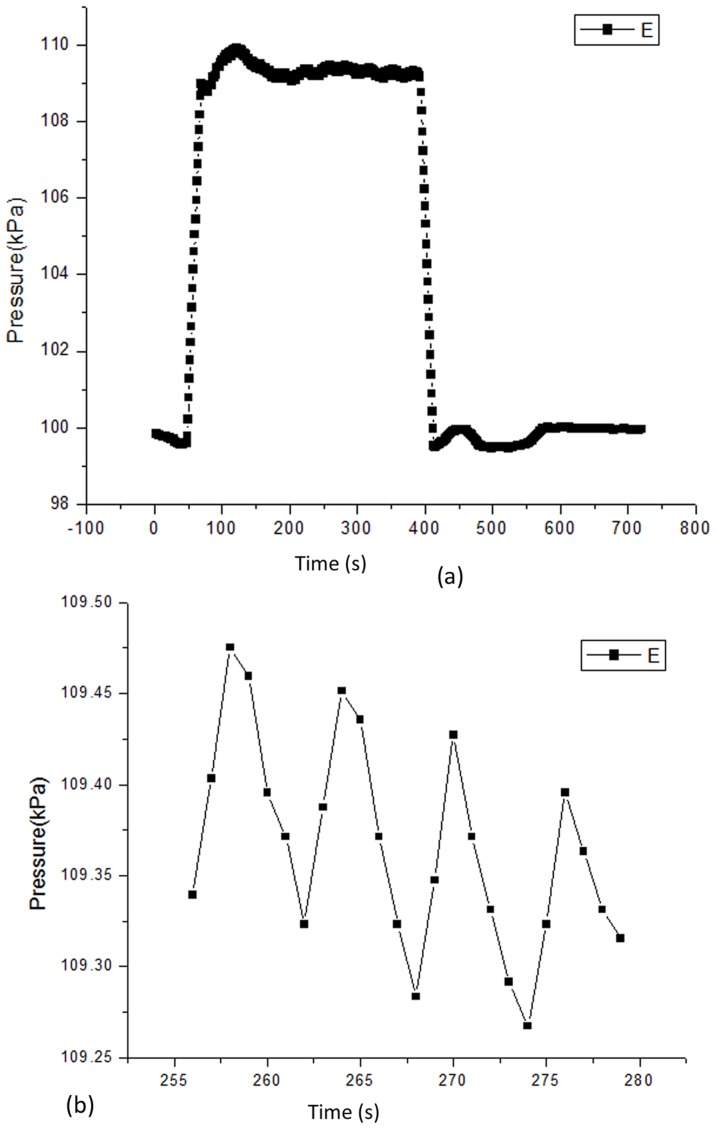
The blood pressure data obtained from the experiment. (a) Units of optical fiber pressure sensor: the vertical axis represents pressure with units of kPa and the horizontal axis represents time with units of *s*. (b) An expanded view of a section of the high pressure curve.

In the experiment, the body temperature of the rabbits was 37°C, and the heart rate was184 beats per minute. The data obtained from the experiment are shown in Fig. 5, the high-pressure portion represents the pressure curve from the measurement of the sensor in the blood vessel, and the low-pressure portion represents the pressure curve when the sensor was removed from the blood vessel. Fig. 5-b presents an expanded view of a section of the high pressure curve in Fig. 5-a.

### 5 Results of rabbit blood fat and routine blood examination

The blood viscosity examination indicated that the blood viscosity of each rabbit was normal; the red cell morphology and rheology status were also normal (Table 1).

**Table 1:** Analysis of the level of rabbit blood fat, hemoglobin, and hemamoeba.

Test items (n = 8)	test results (mol/L)
Triglyceride	0.48±0.09
Cholesterol	1.93±0.33
High density lipoprotein	0.89±0.09
Low density lipoprotein	1.10±0.21
Hemoglobin	132.75±15.47
White blood cells	6.86±2.57
Red blood cell	6.61±0.85
Platelet	392.87±99.67

### 6 Blood pressure results determined using a physiological recorder

The systolic pressure and diastolic pressure results obtained using the physiological recorder were analyzed. The blood pressures of the left and right carotid arteries were not significantly different.

### 7 Blood pressure results determined using optical fiber and Doppler ultrasound

**Figure 6:**
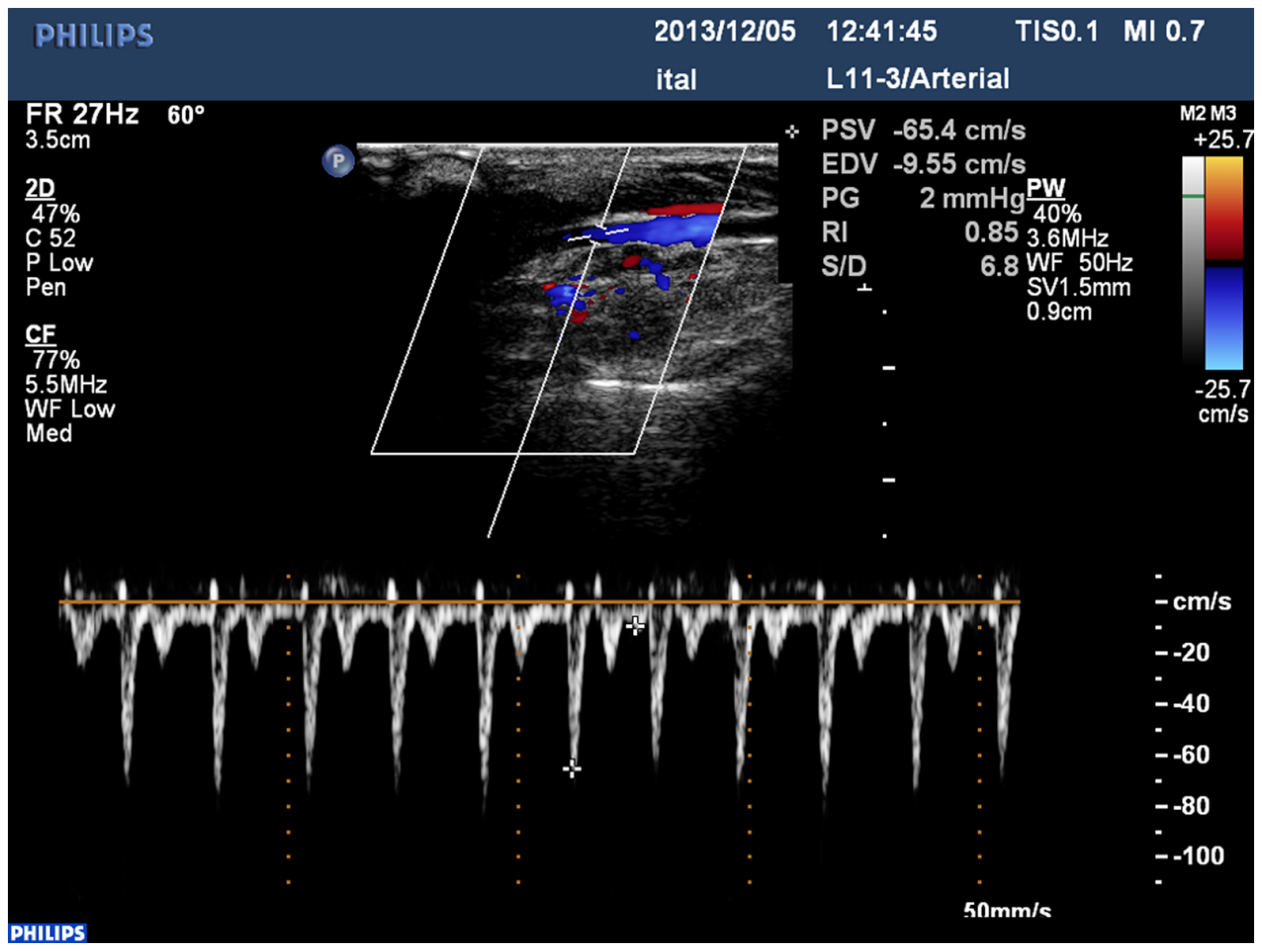
Results determined by color Doppler ultrasound.

The blood pressure of rabbits determined using the optical fiber in the carotid arteries was analyzed. The blood pressure values were not significantly different between the left and right carotid arteries. The results of PSV, EDV, RI, s/d, and PG determined by ultrasound are shown in Fig. 6 and Table 2.

**Table 2:** Determination results of rabbit carotid artery by color Doppler ultrasound.

Test items (n = 8)	test results by color Doppler ultrasound, Left carotid artery	test results by color Doppler ultrasound, right carotid artery
The vessel diameter	2.31±0.15	2.28±0.21*
The peak systolic velocity (PSV)	46.52±11.45	49.13±13.08*
end diastolic velocity (EDV)	9.60±1.42	9.54±1.41*
resistance index (RI)	4.79±1.47	5.16±1.21*
systolic and diastolic flow velocity ratio (s/d)	0.79±0.04	0.79±0.05*
pressure gradient (PG)	1.13±0.35	1.25±0.46*

^*^ P>0.05 compared with the Left carotid artery.

### 8 Correlation of mean blood pressure values determined from blood flow using optical fiber sensor with blood constituents

The mean blood pressure values determined from blood flow using the optical fiber sensor with blood constituents were positively related to the total cholesterol level, low-density lipoprotein level, number of red blood cells, and hemoglobin level, with correlation coefficients of 0.033, 0.129, 0.358, and 0.373, respectively. The blood pressure values exhibited no obvious relationship with the number of white blood cells and high-density lipoprotein levels and had a negative relationship with triglyceride levels and the number of blood platelets, with correlation coefficients of −0.031 and −0.839, respectively.

### 9 Comparison of three methods measuring blood pressure values

**Figure 7:**
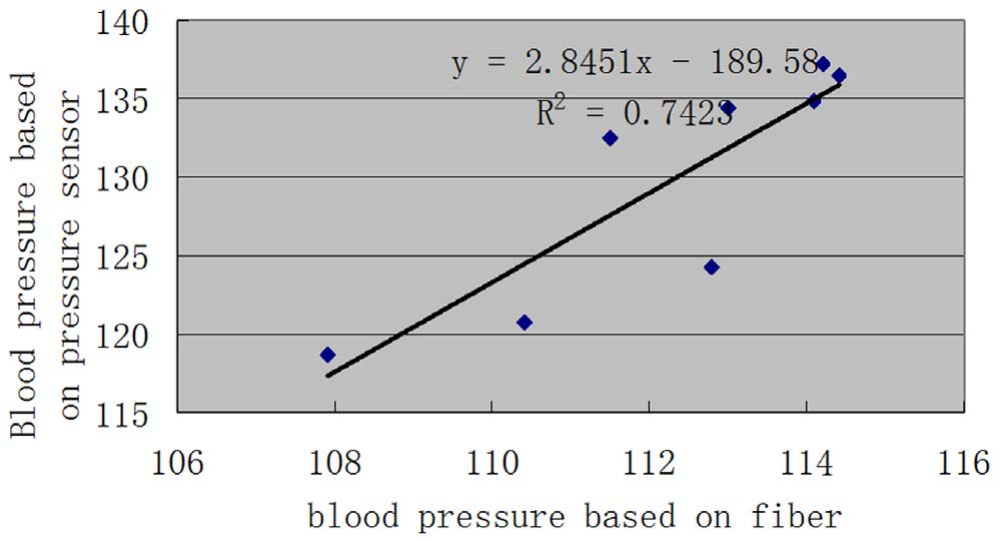
Correlation regression analysis of blood pressure based on the optical fiber sensor and pressure sensor.

The highest position relationship was observed between the optical fiber sensor and the pressure sensor, with a correlation coefficient of 0.86. The optical fiber and PSV determined through ultrasound were positively related, with a correlation coefficient of 0.66; the correlation coefficient between PSV determined using ultrasound and blood pressure determined by the pressure sensor was 0.48. The blood pressure and inner diameter of the blood vessel determined using the ultrasound and optical fiber sensor method had a negative relationship, with correlation coefficients of −0.312 and −0.407, respectively. The blood pressure values and blood vessel diameter determined using the pressure sensor was not related. Based on further analysis of the blood pressure values determined using the optical fiber and EDV and SI determined by ultrasound, the blood pressure values determined using the optical fiber were negatively related to those obtained using EDV, with a correlation coefficient of −0.106. In addition, the blood pressure values were positively related to RI, with a correlation coefficient of 0.534 (Fig. 7).

## Discussion

The micro-vessel is the lowest structural unit of blood circulation in the human body, accounting for 90% of all vessels inside the body. In-depth research on blood flow is of great physiological and pathological significance. Blood flow is a dynamic process that changes constantly. Vein hemospasia and external indirect tests for blood samples can only reflect information at a specific time point. Given the limitations in methods and technologies of current direct real-time tests of blood, no accurate in vivo real-time analysis method for obtaining blood information exists. Obtaining complete real-time vascular rheology information is therefore impossible. Optical fiber chemical sensors have been a research focus in the biomedical field because they are smart, precise, bendable, and nontoxic. These attributes provide a foundation for acquiring and analyzing hemorheology information and other indexes. In deep vessels, particularly those parts unable to be tested by ultrasound, catheter technology can be utilized to apply the optical fiber to evaluate real-time blood flow velocity [3]–[10]. Utilizing interdisciplinary methods; we can overcome limitations in current research methods to realize the acquisition and analysis of in vivo dynamic blood information based on a new optical fiber sensor system. We also explored the basic principles of blood flow from a comprehensive biological perspective, providing a basis for investigating various early-phase micro-angiopathies and exploring new therapeutic targets.

The envelopment of the optical fiber sensor and acquisition and analysis of specific information in a complex blood and tissue liquid environment are challenges in the development of optical fiber sensors. Thus, in the present study, an optical fiber sensor was designed to acquire blood flow information. The flat and round membrane with a hard center was able to deflect the pressure and magnify it, thereby achieving greater deformation under the same pressure. The nonlinear effect is also highly evident. The optical fiber sensor is designed for use as a flat and round membrane structure that is easy to operate and reliable, can be processed and controlled, and possesses a diameter of 1.4 mm, enabling it to enter small vessels.

The optical fiber sensor was able to display the real-time blood flow velocity of rabbits' carotid arteries and dynamically collect and display blood pressure data. From the optical dynamic determination of blood pressure, we observed that the numbers of red blood cells, hemamoeba, blood platelets, and other blood constituents were related to the blood flow velocity. Correlation and regression analyses indicated that the blood pressure values were positively related to the total cholesterol level, low-density lipoprotein level, number of red blood cells and hemoglobin level. No obvious relationship was observed with the number of hemamoeba and high-density lipoprotein level; a negative relationship was observed with triglycerides and the number of blood platelets. These findings indicate that different blood constituents have different effects on blood flow pressure and other hemorheology parameters, showing the importance of defining therapeutic targets. Notably, a negative correlation was observed with the number of blood platelets, with a correlation coefficient of −0.839, indicating that the number of blood platelets has a clear effect on blood flow pressure and other factors. This interesting result indicates that blood platelets may be an important factor that affects blood flow velocity and other hemorheology indexes and are possibly involved in various disease and health conditions in hemorheology. Moreover, anti-platelet therapy may also be useful for various thrombotic diseases and may further improve blood flow status.

In the comparison of the three methods used in determining blood pressure, the correlation coefficient between blood pressure levels determined by the optical fiber and a traditional pressure sensor was 0.86, that between blood pressure levels tested by the optical fiber and color Doppler ultrasound was 0.66, and that between the ultrasound and the pressure sensor was 0.48, indicating that the blood pressure values determined by the optical fiber were more accurate and sensitive. From the correlation analysis of the color ultrasound results, the correlation coefficient between PSV determined by ultrasound and blood pressure values determined by the pressure sensor was 0.48. PSV determined by ultrasound and the blood pressure values and blood vessel diameter determined by the optical fiber were negatively related, with correlation coefficientsof −0.312 and −0.407, respectively. The blood pressure values and blood vessel diameter determined by the pressure sensor were not related, indicating that using the optical fiber to determine blood flow pressure can also indicate blood vessel diameter and other information.

The massive amount of data concerning dynamic changes within the blood poses a challenge to data analysis. To measure the velocity of dynamic blood flow and analyze it in a physiological manner, the present study achieved a breakthrough in terms of research methods by exploring basic methods for obtaining and analyzing blood flow data in vivo via a fiber sensor. Thus, the micro-sensor designed in the present study based on fiber Bragg grating can be used to conduct real-time in vivo determination of blood flow average pressure. This technique is noninvasive, nontoxic, and accurate; it can also be used to optimize determination parameters and realize real-time in vivo blood flow monitoring. The novel Fabry Perot fiber grating sensor can provide partially real-time vascular theology information (including microvascular information). This technique can be used for detection of various types of abnormal blood flow, especially for evaluation of microvascular disease. Therefore, this technique may be useful for early diagnosis of disorders with abnormal blood rheology, such as atherosclerosis, diabetes, hyperlipidemia, coronary heart disease, and peripheral vascular disease, and will also provide a new method for the study of vascular and blood flow diseases.

### The difference compared with other fiber optic pressure sensors

Nesson et al. [11] used polymer, metal film as the sensitive film and a protection layer. Our sensor adopted the isolation groove and the hardtop center. So the structure, production of the program and the results were all different. In Totsu et al. study [12], the material of cavity wall they used was polyimide organic material, while we used the glass material. Because the deformation of the diaphragm was detected by the cavity length, so the material of the cavity wall has a large influence on the stability and precision of the sensor. Wang et al published two papers in year of 2005 and 2006 [13]–[14]. The multimode optical fiber corrosion was used as collimation cavity, and the fused silica miniature was used as the diaphragm. While was different from our program based on MEMS. In the study of MacPherson et al [15], The method they formed the F-P cavity interferometer was the optical fiber inserted into the zirconia collimator, the tail sealing glue or welding. Carefulness was needed in adjusting the distance between the fiber and the diaphragm, which is not easy to batch control. In addition, the fiber and the bonding quality between zirconia quasi straight pipes will long-term effects of spacing between the optical fiber and the diaphragm. In the study of Olson et al [16], they used the organic matters adhered to form the pressure sensitive film, while in our paper we adopted monocrystal silicon as the diaphragm. So both the material and production scheme are not the same. Meanwhile, the sensitive materials lead to range of sensitivity and stability will also be different. In Watson's et al paper [17], they used the online laser processing ways to make the sensitive diaphragm, which is different from our scheme.

As for the patent invented by Belleville et al [18], the sensor without isolation groove and a hardtop center, this will certainly influence the performance of the sensor. The structure used in this patent also is different from our fiber sensor. In the study of Cibula et al [19], they adopted corrosion liquid processing of multimode optical fiber to form a cavity. The pressure sensitive film was formed using the surface tension of the liquid, and the production process needed more manual intervention. The material and processing scheme used in this paper is different from our program based MEMS processing scheme. Abeysinghe et al published two papers in year of 2001 and 2002 [20]–[21]. The production method of the pressure sensor was based on a fiber end face processing. Compared with the batch production based on the MEMS of our method, the repetition of this paper will reduce. In the study of Hill et al [22], they used SU-8 photoresist as the diaphragm, which can make higher sensitivity of the F-P sensor. However, the SU-8 photoresist has toxicity, which is not suitable for biological sensor. As for the patent invented by Sherrer et al [23], our sensor used vacuum cavity structure, while the sensor of this patent's was non vacuum cavity structure. So it cannot measure the negative pressure.

The limitations of the present study include potential damage caused by the process of inserting the sensor in to the arteries, the limited information collected by the optical fiber sensor, and the need for further improvement of optical fiber function to realize the acquisition and analysis of in vivo real-time blood flow information including blood flow velocity, blood flow constituents, and other data.
